# Overexpression of long non‐coding RNA ANRIL promotes post‐ischaemic angiogenesis and improves cardiac functions by targeting Akt

**DOI:** 10.1111/jcmm.15343

**Published:** 2020-05-13

**Authors:** Qun Huang, Miao Pan, Ji‐Peng Zhou, Fei Yin

**Affiliations:** ^1^ Departmen of Pediatrics Xiangya Hospital Central South University Changsha China; ^2^ Department of Child Health Care Hunan Provincial Maternal and Child Health Care Hospital Changsha China; ^3^ Department of Geriatric Medicine Xiangya Hospital Central South University Changsha China; ^4^ National Clinical Research Center for Geriatric Disorders Xiangya Hospital Central South University Changsha China

**Keywords:** Akt, Angiogenesis, ANRIL, Long non‐coding RNA (lncRNA), Myocardial infarction

## Abstract

Angiogenesis is critical for re‐establishing the blood supply to the surviving myocardium after myocardial infarction (MI). Long non‐coding RNA ANRIL (lncRNA‐ANRIL) has been reported to regulate endothelial functions in cardiovascular diseases. This study was to determine the role of lncRNA‐ANRIL in Akt regulation and cardiac functions after MI. Human umbilical vein endothelial cells (HUVECs) were exposed to oxygen‐glucose deprivation (OGD) to mimic in vivo ischaemia. The MI model in mice was induced by ligating left anterior descending coronary artery. OGD remarkably decreased lncRNA‐ANRIL expression level, reduced the phosphorylated levels of Akt and eNOS proteins, and inhibited NO release and cell viability, which were duplicated by shRNA‐mediated gene knockdown of lncRNA‐ANRIL. Conversely, all these effects induced by OGD were abolished by adenovirus‐mediated overexpression of lncRNA‐ANRIL in HUVECs. Further, OGD impaired cell migrations and tube formations in HUVECs, which were reversed by lncRNA‐ANRIL overexpression or Akt up‐regulation. RNA immunoprecipitation analysis indicated that the affinity of lncRNA‐ANRIL to Akt protein was increased in OGD‐treated cells. In animal studies, adenovirus‐mediated lncRNA‐ANRIL overexpression increased the phosphorylated levels of Akt and eNOS, promoted post‐ischaemic angiogenesis and improved heart functions in mice with MI surgery. LncRNA‐ANRIL regulates Akt phosphorylation to improve endothelial functions, which promotes angiogenesis and improves cardiac functions in mice following MI. In this perspective, targeting lncRNA‐ANRIL/Akt may be considered to develop a drug to treat angiogenesis‐related diseases.

## 1 INTRODUCTION

Myocardial infarction (MI) is the leading cause of death in the world.^1^ Angiogenesis is critical for re‐establishing the blood supply to the surviving myocardium after myocardial infarction (MI) and, consequently, to the recovery of cardiac functions.^2^ Angiogenesis depends on cell proliferation, migration and capillary tubulogenesis in endothelial cells.^3^, ^4^ However, the molecular mechanism of angiogenesis remains largely unknown.

Previous studies have indicated that activation of PI3K‐Akt‐dependent signalling improved cardiac functions, reduced infarct size and decreased myocardial apoptosis following MI.^5^ Akt is a serine/threonine kinase regulating essential cellular functions including survival, proliferation, metabolism and patterned gene expression in vascular homeostasis and angiogenesis.^6^ Many of the angiogenic functions attributed to vascular endothelial growth factor are mediated by intracellular activation of Akt signalling.^7^

Long non‐coding RNA (LncRNA) plays important regulatory roles in multiple cellular functions such as epigenetic regulation, cell cycle control, transcription, translation, splicing and cell differentiation mediated by RNA‐RNA, RNA‐DNA or RNA‐protein interactions.^8^, ^9^ The ANRIL gene encodes a 3.8 kb lncRNA which consists of 19 exons, spans over 126 kb, and is highly expressed in vascular cells.^10^ Many studies with human samples showed that the expression level of ANRIL was associated with cardiovascular disease risk.^11^, ^12^, ^13^ Recently, Hyosuk Cho et al reported that lncRNA‐ANRIL regulates endothelial cell activities associated with coronary artery disease by up‐regulating several genes in endothelial cells.^14^ LncRNA‐ANRIL also protects against oxygen and glucose deprivation (OGD)‐induced injury in PC‐12 cells.^15^ However, whether lncRNA‐ANRIL regulates the functions of endothelial cells in angiogenesis after ischaemia is not well‐studied.

Therefore, we hypothesized that lncRNA‐ANRIL may function as a regulator of Akt in endothelial cells. Our results revealed that ischaemia via reduction of lncRNA‐ANRIL down‐regulates Akt/eNOS signalling to impair angiogenesis and cardiac functions. In this perspective, targeting lncRNA‐ANRIL may be an attractive strategy to improve the prognosis of patients with ischaemia‐associated diseases.

## 2 MATERIALS AND METHODS

A full description of materials and methods used, including reagents, animals, cell culture, generations of adenovirus and infections, RNA immunoprecipitation (RIP) assay, animal experimental protocol, evaluation of cell viability, cell migrations, the in vitro model of oxygen‐glucose deprivation (OGD), detection of nitric oxide (NO), eNOS activity assay, the model of MI in mice, echocardiography, capillary density, in vitro tube formation assay, Western blot analysis, real‐time PCR and statistical analysis, can be found in the online‐only Data Supplements.

### 2.1 Animal experimental protocol

Male C57B16 mice (8‐12 weeks old, 25 ± 5 g) were purchased from Hua‐Fu‐Kang Animal Company (Beijing, China). All animals were housed in temperature‐controlled cages with a 12‐hour light‐dark cycle. The surgery of MI was operated by ligation of left anterior descending coronary artery (LADCA) under anaesthesia as described previously.^16^ This study was carried out in strict accordance with the recommendations in the Guide for the Care and Use of Laboratory Animals of the National Institutes of Health. The animal protocols were reviewed and approved by the Animal Care and Use Committees of Central South University.

### 2.2 Echocardiography

As described previously,^17^ echocardiography with standard parasternal and apical views was conducted in the left lateral recumbent position. Systolic or diastolic left ventricular internal diameter (sLVID or dLVID), ejection fraction (EF) and fractional shortening (FS) were calculated.

### 2.3 Cell culture

As described previously,^18^ human umbilical vein endothelial cells (HUVECs) were purchased from Cascade Biologics (Portland, OR) and grown in endothelial basal medium (Clonetics Inc Walkersville, MD). In all experiments, cells were between passages 3 and 8. All cells were incubated at 37°C in a humidified atmosphere of 5% CO_2_ and 95% air.

### 2.4 The in vitro model of oxygen‐glucose deprivation (OGD)

OGD was carried out as described previously.^19^ Briefly, cells were placed in a 37°C anaerobic chamber with O_2_ tension at 1.5%. Cells were washed 3 times and incubated with glucose‐free balanced salt solution that had been deoxygenated by 10 minutes with nitrogen. Control wells were washed and incubated with standard (non‐deoxygenated) balanced salt solution containing 5 mM glucose. pH was maintained between 7.2 and 7.4.

### 2.5 RNA immunoprecipitation (rip) assay

The Magna kit was used for RIP assay as described previously.^20^ Briefly, whole‐cell lysates were incubated at 4°C overnight with magnetic protein A/G beads pre‐treated with 5 μg IgG or Akt antibody. Beads were washed and incubated with proteinase K buffer; then, RNA was isolated from immunoprecipitates, and cDNA was synthesized.

### 2.6 Statistical analysis

All quantitative data are reported as mean ± SEM and were analysed using a one‐way ANOVA. Bonferroni corrections were applied to multiple comparisons. Comparisons between two groups were analysed by unpaired Student's *t* test. *P* < 0.05 was considered as significant.

## 3 RESULTS

### 3.1 OGD decreases lncRNA‐ANRIL expression and induces endothelial dysfunction in HUVECs

**Figure 1:**
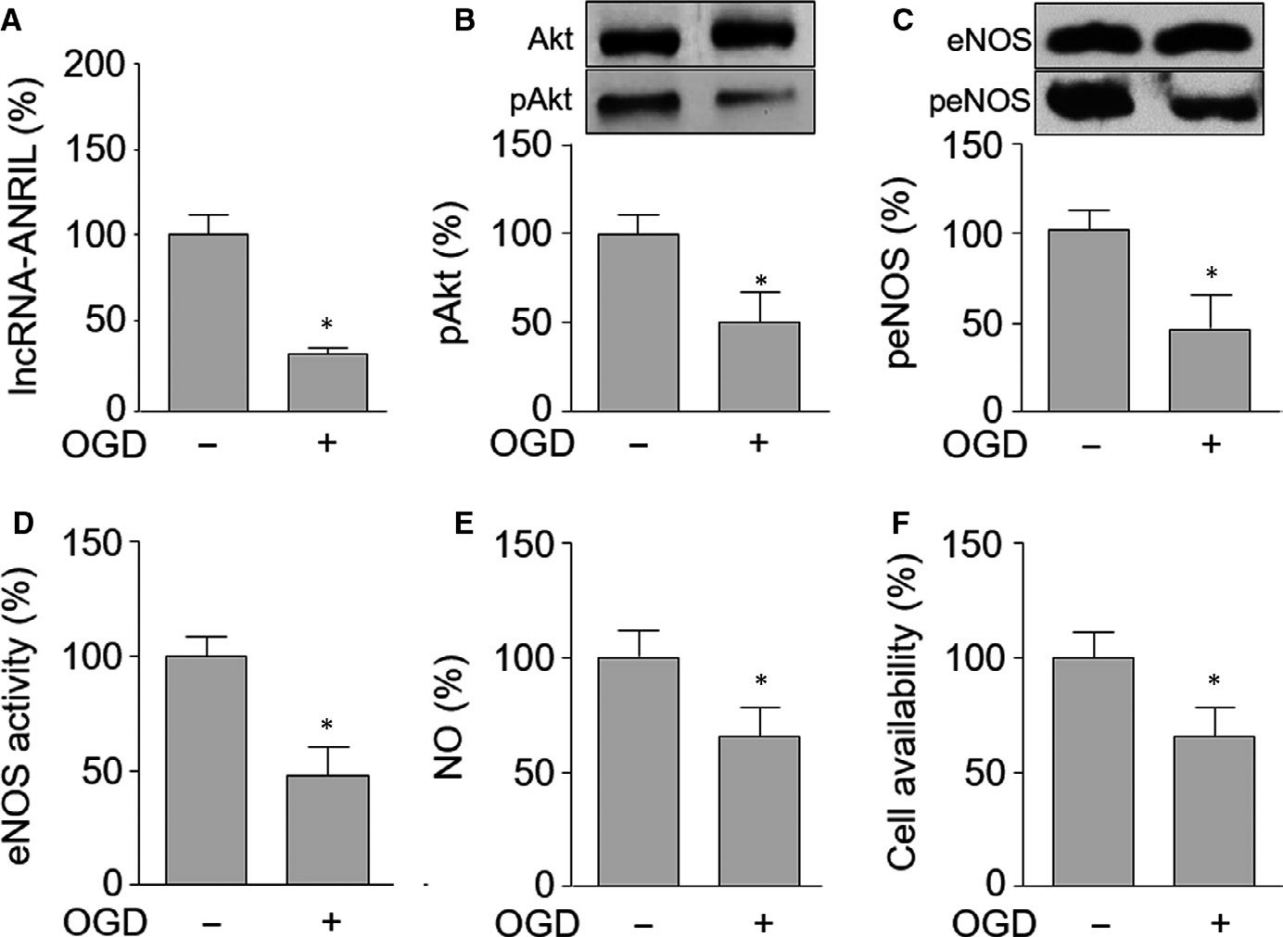
Oxygen‐glucose deprivation (OGD) decreases gene expression of lncRNA‐ANRIL, reduces the phosphorylated levels of Akt and eNOS proteins, and impairs cellular functions in HUVECs. Cultured HUVECs were exposed to OGD for 6 hours. (A) The lncRNA‐ANRIL level was assessed by real‐time PCR. (B and C) Total cell lysates were subjected to perform Western blot to measure the phosphorylated levels of Akt in B and eNOS in C. (D) The eNOS activity in total cell lysates was assayed by the method of L‐[^3^H]citrulline production from L‐[^3^H]arginine. (E) Intracellular nitric oxide (NO) productions were determined by assaying DAF fluorescence. (F) Cell viability was measured by MTT assay. N is 5 in each group. *P < 0.05 vs control

Previous studies have reported that lncRNA‐ANRIL regulates endothelial cell function^21^ and endothelial cell is a key cell contributing to ischaemia‐induced angiogenesis.^3^, ^4^ Thus, we firstly determined the effects of ischaemia on lncRNA‐ANRIL gene expression in cultured HUVECs. The model of OGD was used to mimic ischaemia in vivo. As shown in Figure 1A, OGD dramatically decreased lncRNA‐ANRIL expressional level, compared to cells without OGD, indicating ischaemia may down‐regulate lncRNA‐ANRIL gene expression.

### 3.2 OGD decreases AKT/ENOS signalling in HUVECs

Akt has been identified as an eNOS upstream kinase,^22^ and the Akt/eNOS signalling is critical to endothelial cell‐mediated angiogenesis.^6^ We next measured the phosphorylated levels of Akt at serine 473 and eNOS at serine 1179, which represent their activities as described previously.^23^, ^24^ As shown in Figure 1B and C, exposure of HUVECs to OGD decreased both Akt and eNOS phosphorylations, similar with other reports.^25^ The inhibition of Akt/eNOS signalling was further confirmed by measuring eNOS activity in Figure 1D. The activity of eNOS was totally reduced in cells treated with OGD.

### 3.3 OGD impairs cellular functions in HUVECs

NO released from eNOS has been considered as endothelial function.^26^, ^27^ Thus, we determined the function of HUVECs by measuring NO productions. As shown in Figure 1E, OGD significantly reduced NO productions and inhibited cell viabilities, compared to control cells. The impaired cellular functions of HUVECs were also confirmed by measuring cell viabilities (Figure 1F). OGD inhibited cell viabilities, as determined by MTT, compared to control cells without OGD. Taking these data, it suggests that ischaemia may inhibit lncRNA‐ANRIL/Akt/eNOS to impair the functions of endothelial cells.

### 3.4 Overexpression of lncRNA‐ANRIL abolishes OGD‐reduced AKT and eNOS phosphorylations in huvecs

**Figure 2:**
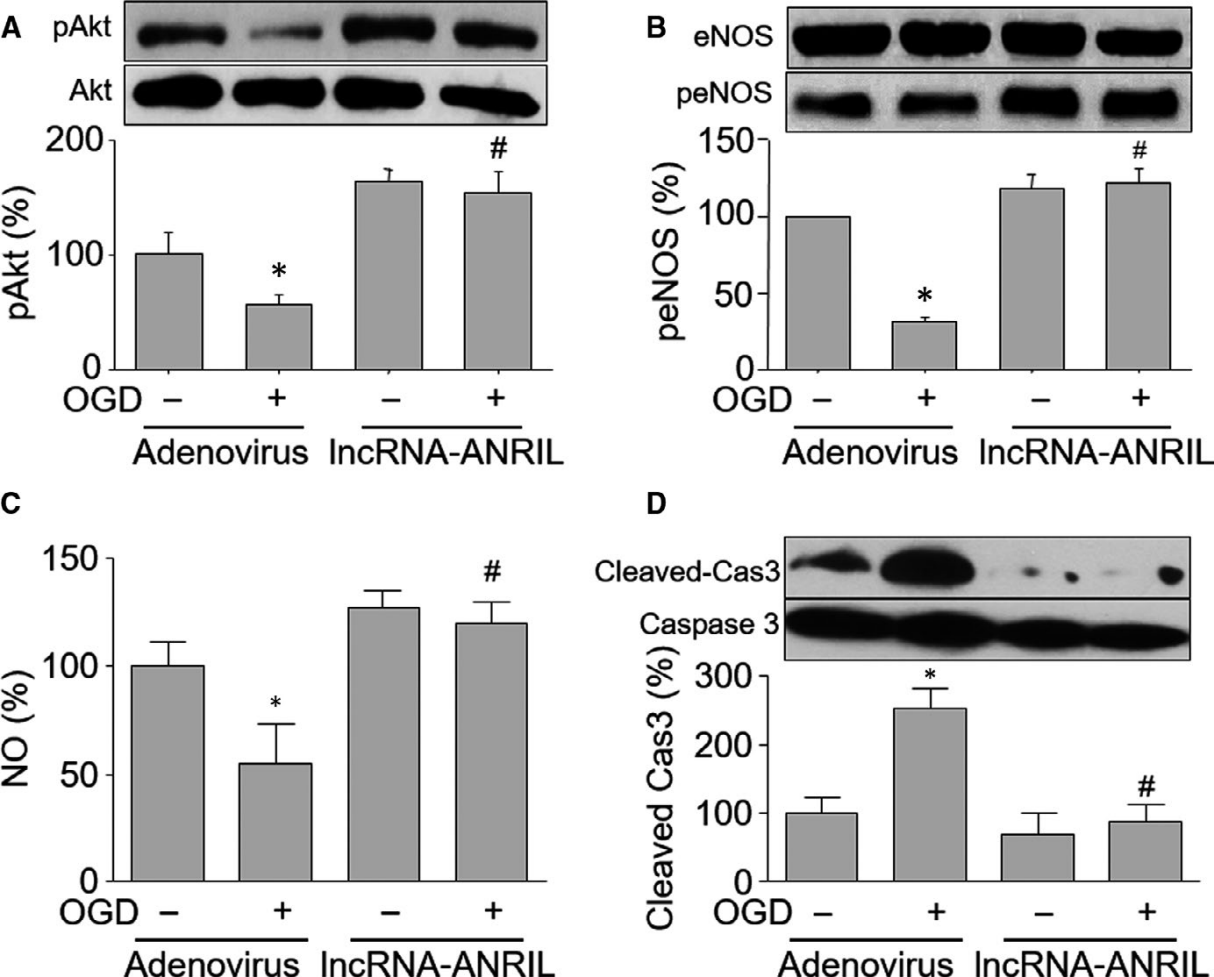
Adenovirus‐mediated lncRNA‐ANRIL overexpression abolishes OGD‐induced reductions of Akt and eNOS phosphorylations in HUVECs. Cultured HUVECs were infected with adenovirus expressing lncRNA‐ANRIL for 48 hours and then treated with OGD for 6 hours. (A and B) Total cell lysates were subjected to perform Western blot to measure the phosphorylated levels of Akt in A and eNOS in B. (C) Intracellular NO productions were determined by DAF fluorescence. (D) The levels of cleaved caspase 3 and total caspase 3 were measured by Western blot. N is 5 in each group. ^*^ *P* < 0.05 vs adenovirus alone. ^#^ *P* < 0.05 vs adenovirus plus OGD

To investigate whether OGD via lncRNA‐ANRIL down‐regulation inhibits Akt/eNOS signalling in HUVECs, we infected cells with adenovirus expressing lncRNA‐ANRIL and then treated cells with OGD. As indicated in Figure 2A and B, OGD completely reduced both Akt and eNOS phosphorylations in HUVECs infected with adenovirus vector, but not in cells with overexpressed lncRNA‐ANRIL. Accordingly, adenovirus‐mediated lncRNA‐ANRIL overexpression reversed NO productions (Figure 2C) and the level of cleaved caspase 3 (Figure 2D) in HUVECs treated with OGD. These data demonstrated that lncRNA‐ANRIL is involved in OGD‐induced Akt/eNOS inactivation in endothelial cells.

### 3.5 Gene knockdown of lncRNA‐ANRIL reduces the phosphorylated levels of AKT and eNOS proteins in HUVECs

To further confirm the role of lncRNA‐ANRIL in OGD‐induced inactivation of Akt‐eNOS signalling, we infected cells with lncRNA‐ANRIL shRNA to silence lncRNA‐ANRIL function. As shown in Figure S1A and S1B, lncRNA‐ANRIL shRNA remarkably decreased both Akt phosphorylation and eNOS phosphorylation, compared to HUVECs infected with adenovirus vector alone, demonstrating that lncRNA‐ANRIL is an upstream regulator of Akt in HUVECs.

### 3.6 LncRNA‐ANRIL deficiency mimics the effects of OGD on cell availability of HUVECs

We also detected the effects of lncRNA‐ANRIL gene knockdown on cellular functions of HUVECs. Similar to OGD, lncRNA‐ANRIL shRNA noticeably decreased NO productions (Figure S1C) and impaired cell viabilities (Figure S1C), compared to HUVECs infected with adenovirus alone. These data imply that lncRNA‐ANRIL may mediate the effects of OGD on cellular functions.

### 3.7 OGD via down‐regulation of lncRNA‐ANRIL inhibits cell migrations in HUVECs

**Figure 3:**
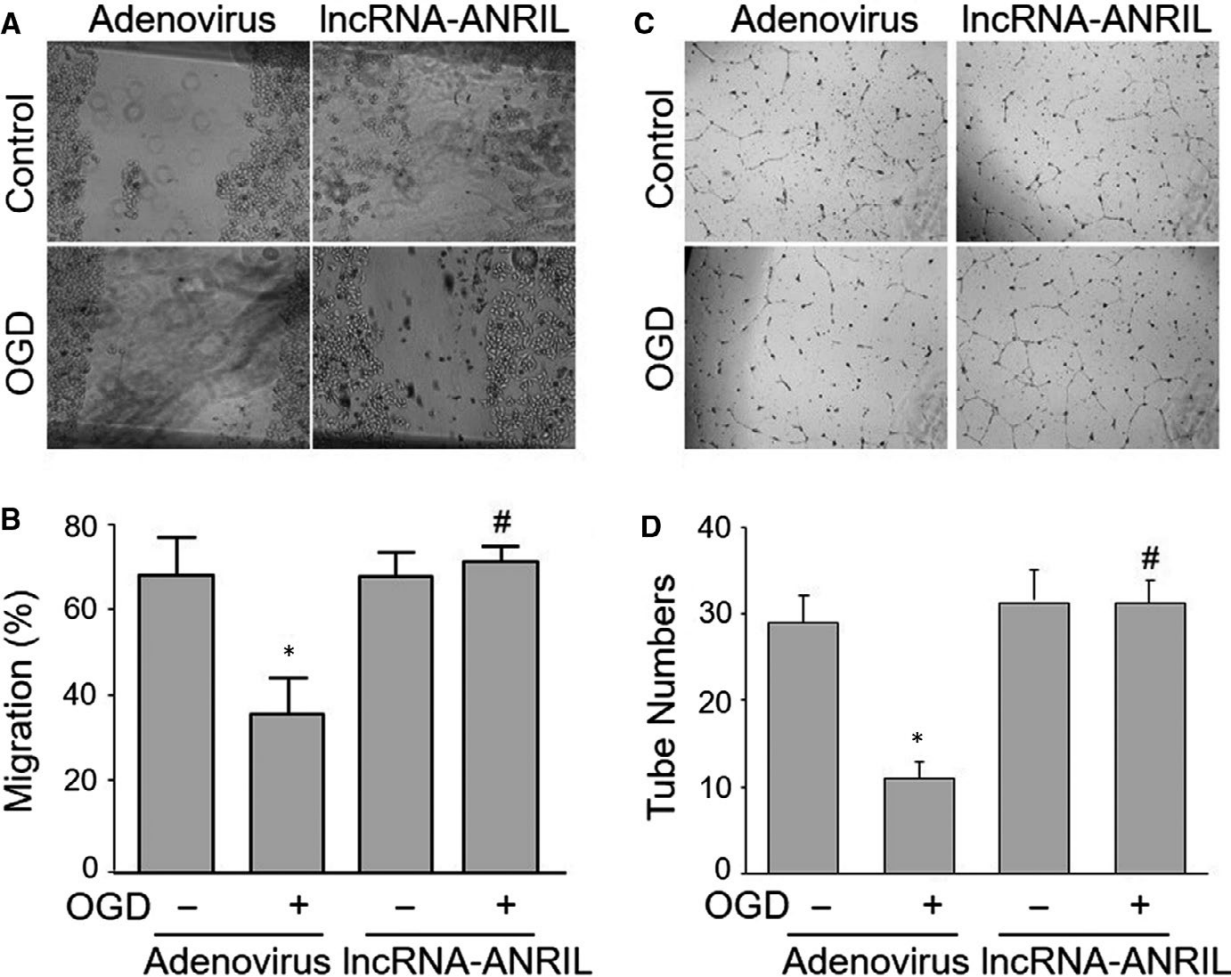
Overexpression of lncRNA‐ANRIL ablates OGD‐induced impairments of cell migration and tubulogenesis in HUVECs. Cultured HUVECs were infected with adenovirus expressing lncRNA‐ANRIL for 48 hours and then treated with OGD for 6 hours. (A and B) Cell migration was determined by scratch test. Migration rate was calculated in the 3rd day after scratch. The representative pictures are shown in A. Quantitative analysis is shown in B. (C and D) Tubulogenesis of HUVECs was determined by tube formation test. The representative pictures of tube formations are presented in C, and quantitative analysis was performed by calculating tube numbers per scope in D. N is 5 in each group. ^*^ *P* < 0.05 vs adenovirus alone. ^#^ *P* < 0.05 vs adenovirus plus OGD

Cell migration is critical to the post‐ischaemic angiogenesis.^4^ We next examined whether overexpression of lncRNA‐ANRIL reversed OGD‐inhibited cell migrations in HUVECs. As shown in Figure 3A and B, OGD inhibited the migration rates in HUVECs infected with adenovirus vector, but not in cells infected with adenovirus harbouring lncRNA‐ANRIL cDNA. These findings prove that down‐regulation of lncRNA‐ANRIL is crucial to the cell migrations impaired by OGD.

### 3.8 Up‐regulation of lncRNA‐ANRIL bypasses OGD‐impaired tubulogenesis in HUVECs

Tube formation is also a vital step in endothelial cell‐mediated angiogenesis.^4^ Therefore, we examined whether lncRNA‐ANRIL overexpression reversed OGD‐impaired tube formation in HUVECs. As shown in Figure 3C and D, OGD inhibited the tube formation of HUVECs infected with adenovirus alone, while the effects of OGD on tubulogenesis were bypassed if cells were infected with adenovirus expressing lncRNA‐ANRIL cDNA. Collectively, these data suggest that OGD via down‐regulation of lncRNA‐ANRIL inhibits tubulogenesis in endothelial cells.

### 3.9 Up‐regulation of AKT rescues OGD‐impaired cell migrations and tubulogenesis in HUVECs

Akt has been reported to promote ischaemia‐induced angiogenesis.^28^ We next examined whether overexpression of Akt reversed OGD‐inhibited cell viability, migration and tubulogenesis in HUVECs. As shown in Figure S2A, similar to lncRNA‐ANRIL overexpression (Figure 2D), adenovirus‐mediated Akt overexpression maintained cell viabilities in HUVECs treated with OGD. Further, OGD inhibited the migration rates (Figure S2B) and tube formation (Figure S2C) in HUVECs infected with adenovirus vector, but not in cells infected with adenovirus expressing Akt cDNA. These findings, hence in combination, prove that Akt activation is required to improve cell migrations and tubulogenesis impaired by OGD.

### 3.10 OGD decreases the affinity of lncRNA‐ANRIL to AKT protein in HUVECs

**Figure 4:**
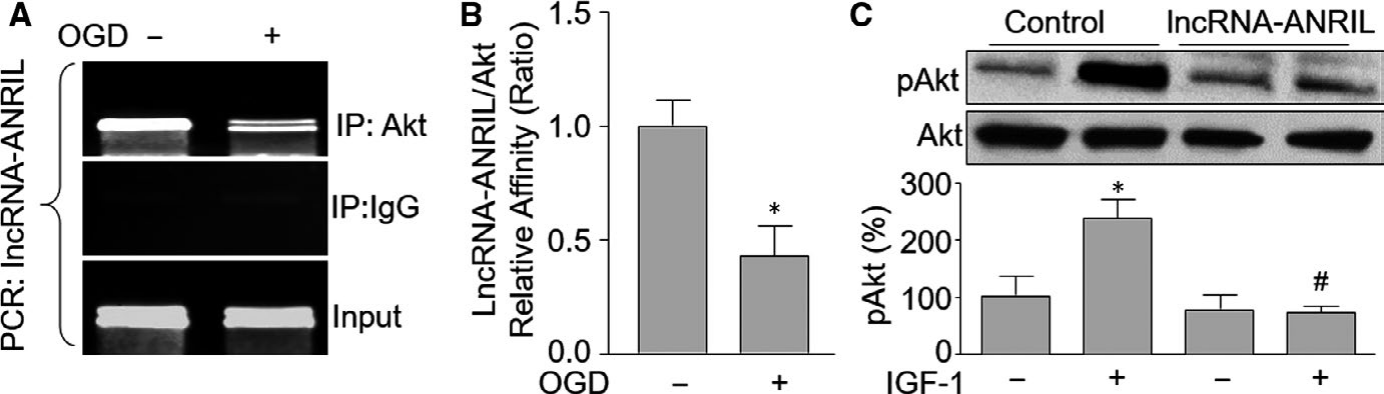
LncRNA‐ANRIL regulates Akt phosphorylation by binding to Akt protein directly. (A and B) Cultured HUVECs were exposed to OGD for 6 hours. Cells were subjected to detect the binding of lncRNA‐ANRIL to Akt protein by using RNA immunoprecipitation assay in A. Quantitative analysis of the affinity between lncRNA‐ANRIL and Akt protein was performed in B. N is 5 in each group. ^*^ *P* < 0.05 vs control. (C) Cultured HUVECs were infected with adenovirus expressing lncRNA‐ANRIL shRNA or control shRNA for 48 hours followed by treatment with IGF‐1 (100 ng/ml) for 24 hours. Total cell lysates were subjected to perform Western blot analysis of phosphorylated levels of both Akt and eNOS proteins in C. N is 5 in each group. ^*^ *P* < 0.05 vs control shRNA. ^#^ *P* < 0.05 vs control shRNA plus IGF‐1

Protein function can be post‐translationally regulated by protein‐RNA interactions.^29^, ^30^ Thus, we hypothesized that lncRNA‐ANRIL may interact Akt protein to regulate Akt phosphorylation in endothelial cells. To test this notion, we performed RIP analysis to determine the affinity between lncRNA‐ANRIL and Akt protein by pulldown of Akt in HUVECs after OGD treatment. As observed in Figure 4A and B, lncRNA‐ANRIL was positively amplified in samples from cells following RIP with Akt primary antibody but not with control IgG, implying that the positive amplification of lncRNA‐ANRIL is specific to Akt in endothelial cells under resting condition. Importantly, OGD solidly decreased the binding of lncRNA‐ANRIL to Akt protein. These data support that lncRNA‐ANRIL may regulate Akt phosphorylation through interacting with Akt protein.

### 3.11 LncRNA‐ANRIL is essential for AKT activation in HUVECs treated with IGF‐1

To further verify this concept that lncRNA‐ANRIL is a regulator of Akt phosphorylation in HUVECs, we down‐regulated lncRNA‐ANRIL in HUVECs by adenovirus‐mediated shRNA and then treated cells with IGF‐1, which activates Akt in endothelial cells.^31^ As shown in Figure 4C, IGF‐1 noticeably increased Akt phosphorylation in HUVECs infected with adenovirus expressing control shRNA, but not in cells infected with adenovirus expressing lncRNA‐ANRIL shRNA. In sum, it demonstrates that lncRNA‐ANRIL is required for Akt activation in endothelial cells.

### 3.12 Overexpression of lncRNA‐ANRIL promotes ischaemia‐induced angiogenesis in mice hearts

**Figure 5:**
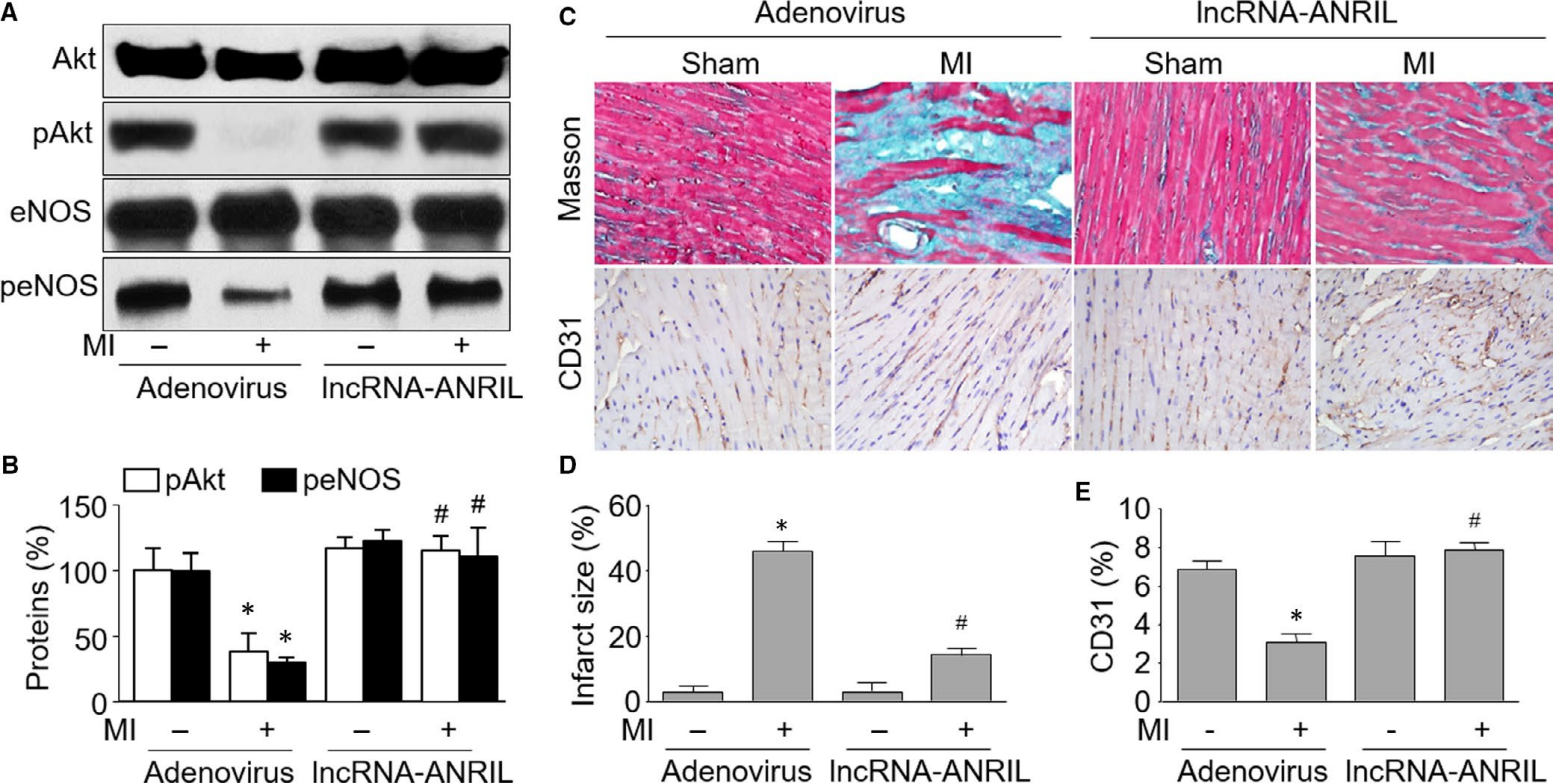
Adenovirus‐mediated overexpression of lncRNA‐ANRIL promotes angiogenesis in post‐ischaemic myocardium in mice. The protocol and experimental designs are described in Methods and Figure S3A. (A and B) Tissues of hearts were subjected to perform Western blot analysis of the phosphorylated levels of both Akt and eNOS proteins. The representative pictures of Western blot are presented in A, and quantitative analysis is performed in B. (C) Representative images showing capillary density by IHC analyses of CD31 and cardiac remodelling by Masson's staining in ischaemic hearts from mice. (D) Quantitative analyses of infarct size. (E) Quantitative analyses of CD31. N is 10‐15 in each group. *P < 0.05 vs sham plus adenovirus. ^#^ *P* < 0.05 vs adenovirus plus MI

Angiogenesis is a key regenerative event to re‐establish blood supply and repair infarcted area after MI in heart.^2^ Next, we determined whether lncRNA‐ANRIL was critically involved in the angiogenic response in vivo. To this end, mice were subjected to induce MI by LADCA ligation for 2 weeks (Figure S3A). Adenovirus‐mediated gene overexpression through in situ injection was applied to mice 2 weeks prior to MI surgery. The efficiency of the delivery system was assessed in Figure S3B and S3C by measuring the levels of lncRNA‐ANRIL using FISH and real‐time PCR. As shown in Figure 5A and B, lncRNA‐ANRIL overexpression increased the levels of Akt phosphorylation and eNOS phosphorylation in hearts isolated from mice following MI, supporting the notion that lncRNA‐ANRIL is an upstream regulator of Akt in vivo.

Capillary densities were assessed in ischaemic hearts on the 14th post‐operative day by staining with antibodies against CD31, which is a biomarker of newborn vessel.^32^ As indicated in Figure 5C‐E, capillary density exhibited a robust increase in ischaemic hearts from mice with lncRNA‐ANRIL overexpression, compared to mice infected with adenovirus expressing control vector. Accordingly, Masson's staining analysis revealed that up‐regulation of lncRNA‐ANRIL reduced cardiac remodelling in hearts from MI mice, compared with mice expressing vector alone. These data display that lncRNA‐ANRIL up‐regulation is required for ischaemia‐induced angiogenesis in vivo.

### 3.13 Overexpression of lncRNA‐ANRIL improves the recovery of heart functions in mice following MI

**Figure 6:**
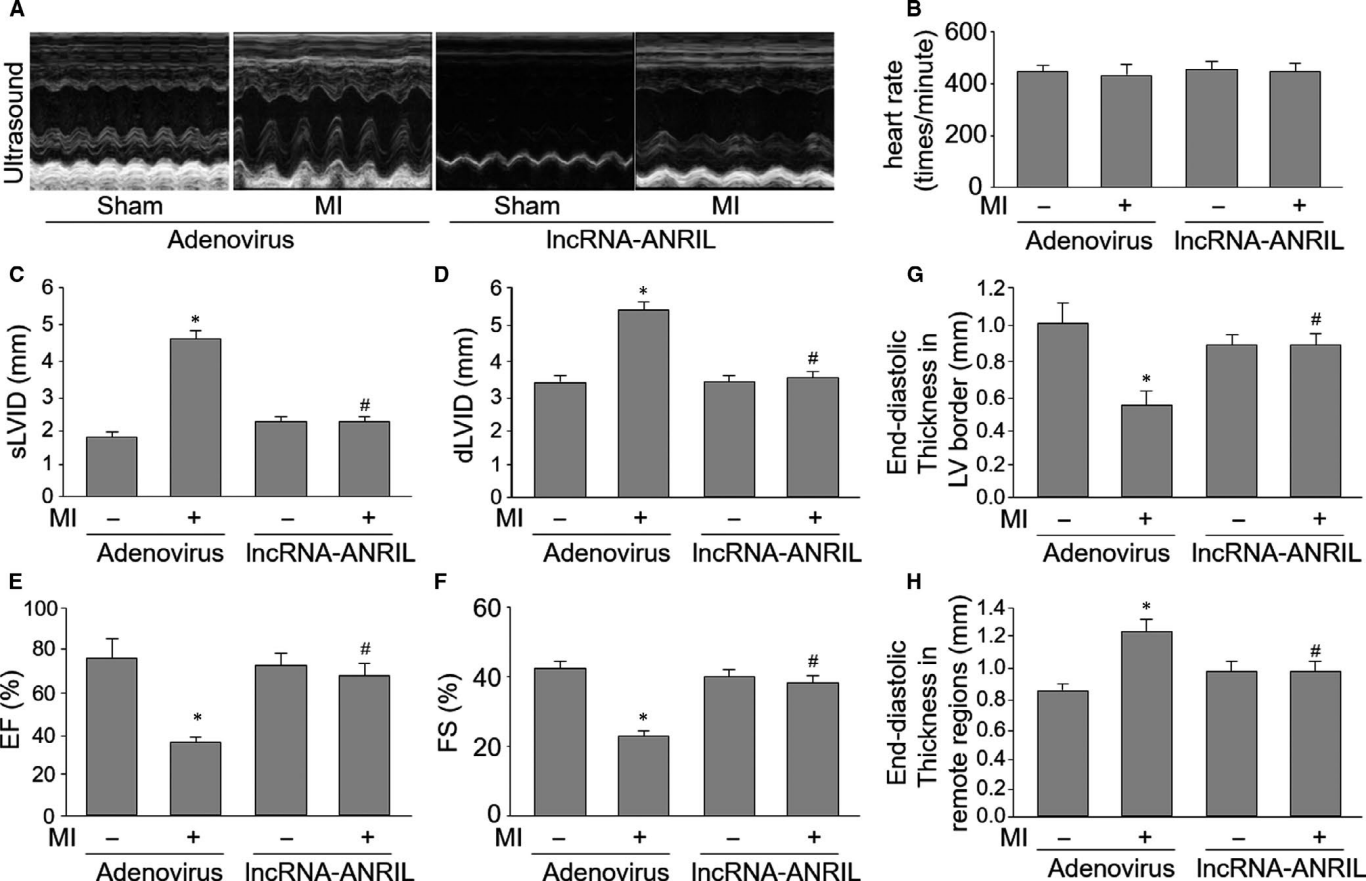
Adenovirus‐mediated gene overexpression of lncRNA‐ANRIL improves cardiac functions in mice following MI. The protocol and experimental designs are described in Supplement Methods and Figure S3A. (A) Functional analysis by echocardiography in mice. (B‐H) Quantitative analyses of heart rate in B, sLVID in C, dLVID in D, EF in E, FS in F, end‐diastolic thickness in LV border in G and end‐diastolic thickness in remote regions in H were performed. N is 10‐15 in each group. *P < 0.05 vs sham plus adenovirus. ^#^ *P* < 0.05 vs adenovirus plus MI

Knowing that lncRNA‐ANRIL/Akt/eNOS pathway is a key mechanism for ischaemia‐induced angiogenesis, we speculated that this signalling would be involved in the impairment of heart functions in mice after MI. To test this notion, we examined heart functions by echocardiographic analysis two weeks after MI surgery in mice. As shown in Figure 6A‐E, two weeks post‐LADCA ligation, in comparison with mice with sham surgery, ischaemia increased sLVID, dLVID and end‐diastolic thickness in remote regions, but decreased FS, EF and end‐diastolic thickness in LV border in mice, consistent with other reports.^33^ By contrast, overexpression of lncRNA‐ANRIL did improve cardiac functions in mice with MI surgery, suggesting that lncRNA‐ANRIL activation contributes to the heart functional recovery after MI.

## 4 DISCUSSION

In the present study, we provided the evidences to determine that lncRNA‐ANRIL is a regulator of Akt in endothelial cells, but also to show that ischaemia via inhibition of lncRNA‐ANRIL/Akt/eNOS pathway impairs endothelial cell functions and cardiac functions. Molecular mechanically, lncRNA‐ANRIL increases Akt function by binding to Akt protein. In mice, overexpression of lncRNA‐ANRIL promotes angiogenesis and improves heart functions. We conclude that lncRNA‐ANRIL up‐regulation is required for Akt activation to promote angiogenesis.

The major discovery of the present project is that lncRNA‐ANRIL regulates Akt phosphorylation in endothelial cells. To the best of our knowledge, this is the first study to identify lncRNA‐ANRIL as a new regulator of Akt activation. This notion is supported by the following evidences: (1) RIP analysis revealed the specific binding of Akt protein with lncRNA‐ANRIL as Akt protein in total cell lysates was purified by using primary Akt antibody but not control IgG; (2) up‐regulation of lncRNA‐ANRIL increased Akt phosphorylation induced by IGF‐1, while lncRNA‐ANRIL down‐regulation reduced Akt phosphorylation; (3) the downstream effector of Akt signalling including eNOS and endothelial functions was also controlled by lncRNA‐ANRIL loss of function or gain of function; (4) overexpression of lncRNA‐ANRIL or Akt produces similar effects on OGD‐impaired cell migration and tubulogenesis. Although our observations provided support on lncRNA‐ANRIL‐dependent regulation of Akt phosphorylation in endothelial cells, the molecular mechanisms by how lncRNA‐ANRIL regulates Akt phosphorylation need to be further investigated.

Another discovery of this project is that ischaemia‐induced angiogenesis is lncRNA‐ANRIL‐dependent. Angiogenesis is a vital process for embryological growth, tissue development and wound healing in damaged tissues.^34^ Angiogenesis requires angiogenic factors, such as VEGF and IGF, to stimulate vessel sprouting and remodelling of the primitive vascular network, which in turn establishes stable and functional blood vessel networks.^35^, ^36^ In response to these angiogenic factors, a common factor is Akt,^7^ which is required for the normal growth of new blood vessels or neovascularization. In this study, we found that lncRNA‐ANRIL functions as a mediator of angiogenesis in heart after ischaemia through Akt. Further, Akt is essential for this process of lncRNA‐ANRIL through RNA‐protein interaction.

An issue needs to be discussed is how lncRNA‐ANRIL regulates Akt phosphorylation. Till now, Akt has been reported to be phosphorylated at two key residues on Thr308 in the activation of the catalytic protein kinase and Ser473 in a C‐terminal hydrophobic motif,^37^, ^38^ which are regulated by PI3K upon extracellular stimuli. Akt signalling is also to be terminated by lipid phosphatases such as PTEN and INPP4B, two critical protein phosphatases function to directly inactivate Akt. In addition, protein phosphatase 2A dephosphorylates Akt Thr308, leading to kinase inactivation.^39^ The PH domain leucine‐rich repeat protein phosphatases were discovered as the physiological Akt Ser473 phosphatases, in which they dephosphorylate Ser473 on specific Akt isoforms.^40^ Our data firstly ascertain that lncRNA‐ANRIL directly regulates Akt phosphorylation by binding to Akt protein. Associated with that Akt is involved in multiple biological functions, such as lipid and glucose metabolisms, inflammation, development, cardiovascular disease and cancer,^41^ identification of lncRNA‐ANRIL as a regulator of Akt not only helps us to understand the molecular mechanism of Akt regulation, but also explores the novel role of lncRNA‐ANRIL in other aspects related to Akt.

Further, another question is how OGD down‐regulates lncRNA‐ANRIL gene expression in endothelial cells. We speculated microRNAs including miR‐133a and miR‐199, as the important regulators of endothelial cell functions, may contribute to lncRNA‐ANRIL repression because these pathways are dysregulated in cardiovascular diseases.^42^, ^43^

In summary, the present study proposes a role of lncRNA‐ANRIL in the tissue response of angiogenesis to ischaemic stress. Specifically, when angiogenesis is induced by ischaemia in tissues, lncRNA‐ANRIL is inactivated by ischaemia, leading to Akt inactivation. Akt suppression serves to maintain low levels of eNOS phosphorylation, which is ultimately not enough for a normal angiogenesis (Figure S4). Further delineation of these proposed mechanisms will be necessary before a complete understanding of this process is achieved.

## CONFLICT OF INTEREST

The author declare that they have no conflicts of interest.

## AUTHOR CONTRIBUTIONS

QH designed and conducted the experiments, and analysed data. MP and JPZ partially performed some experiments. FY designed and performed the experiments, analysed data, wrote the manuscript and convinced the whole project.

## Supporting information

Supplementary Material

## Data Availability

Data availability is upon the request.
