# Supplementary material for: Overexpression of long non‐coding RNA ANRIL promotes post‐ischaemic angiogenesis and improves cardiac functions by targeting Akt
Source: J Cell Mol Med. 2020 May 13;24(12):6860–8. doi: 10.1111/jcmm.15343 (PMC7299705; doi:10.1111/jcmm.15343)
Supplement: Supplementary file 1 — Supplementary Material [file JCMM-24-6860-s001.docx]

**Online Supplements**

**Overexpression of long noncoding RNA ANRIL promotes post-ischemic angiogenesis and improves cardiac functions by targeting Akt**

Qun Huang^1, 2^, Miao Pan^3^, Ji-Peng Zhou^3, 4,*^, Fei Yin^1,*^

^1^Departmen of Pediatrics, Xiangya Hospital, Central South University, Changsha, Hunan 410008, China; ^2^Department of Child Health Care, Hunan Provincial Maternal and Child Health Care Hospital, Changsha, Hunan 410008, China; ^3^Department of Geriatric Medicine, Xiangya Hospital, Central South University, Changsha, Hunan 410008, China; ^4^National Clinical Research Center for Geriatric Disorders, Xiangya Hospital, Central South University, Changsha, Hunan 410008, China

^*^These authors contributed equally to this article.

Correspondence to: Dr. Ji-Peng Zhou, Department of Geriatric Medicine, Xiangya Hospital, Central South University, Changsha, Hunan 410008, China, E-Mail: zhoujipeng616@csu.edu.cn or Dr. Fei Yin, Department of Pediatrics, Xiangya Hospital, Central South University, Changsha, Hunan 410008, China, E-Mail: yinfei@csu.edu.cn.

**Contents**

1. Supplementary Materials and Methods

2. Supplementary Figure Legends S1-S4

**Supplementary Methods and Materials**

**Reagents**

Polyclonal or monoclonal antibodies against Akt, eNOS, pAkt, peNOS, and CD31 were obtained from Cell Signaling Company. Lipofectamine^TM^ Max was from Invitrogen. The kit of eNOS activity assay was from Cell Signaling Company. All drug concentrations were expressed as the final molar concentration in the buffer.

**Cell cultures**

Human umbilical vein endothelial cells (HUVECs) were purchased from Cascade Biologics (Portland, OR) and grown in endothelial basal medium (Clonetics Inc. Walkersville, MD) supplemented with 2% fetal calf serum (FCS) and growth factors, penicillin (100 u/ml), and streptomycin (100 µg/ml). In all experiments, cells were between passages 3 and 8. All cells were incubated at 37°C in a humidified atmosphere of 5% CO_2_ and 95% air. Cells were grown to 70-80% confluent with starvation before being treated with different agents.

**Generation of DNA construct and adenovirus infection to cells or mice**

cDNAs of Akt and lncRNA-ANRIL were purchased from Origene Company. The adenovirus constructions compassing Akt and lncRNA-ANRIL were generated using the AdMax (Microbix) and pSilencer™ adeno 1.0-CMV (Ambion) systems according to the manufacturers’ recommendations. Viruses were packaged and amplified in HEK293A cells and purified using CsCl_2_ banding followed by dialysis against 10 mM Tris-buffered saline with 10% glycerol. Titering was performed on HEK293 cells using the Adeno-X Rapid Titer kit (BD Biosciences Clontech, Palo Alto, CA, USA) according to the manufacturer’s instructions. HUVECs were infected with adenovirus overnight in antibiotics-free medium supplemented with 2% FBS. The cells were then washed and incubated in fresh medium for an additional 12-hour before experiments. For infecting mice, adenovirus was injected via tail vein under pressure in 1 ml of PBS with 7.6 X 10^7^ IFUs of loaded virus. The concentration of DNA was 10 mg/kg.

**RNA quantification**

Total RNA was isolated using a TRIzol-based (Invitrogen) RNA isolation protocol. RNA was quantified by Nanodrop (Agilent Technologies) using an Agilent 2100 Bioanalyzer (Agilent Technologies). Samples required 260/280 ratios of more than 1.8, and sample RNA integrity numbers of more than 9 for inclusion. RNA was reverse transcribed using the TaqMan RNA Reverse Transcription Kit (Applied Biosystems) according to the manufacturer’s instructions. PCR cycling conditions were 95°C for 3 min, 40 cycles of 95°C for 10 sec, 56°C for 10 sec, and 72°C for 10 sec.

**Western blot**

Cells or tissues were homogenized on ice in cell-lysis buffer containing 20 mM Tris-HCl (pH 7.5), 150 mM NaCl, 1 mM Na_2_EDTA, 1 mM EGTA, 1% Triton, 2.5 mM sodium pyrophosphate, 1 mM beta-glycerophosphate, 1 mM Na_3_VO_4_, 1 µg/ml leupeptin, and 1 mM PMSF. Protein samples were solubilized in SDS sample buffer, and 20 µg of protein was separated by SDS-PAGE using 8-10% polyacrylamide gels, transferred to nitrocellulose membranes. Entire sheets of Hybond-ECL membranes containing transferred proteins were incubated firstly in 5% non-fat dry milk for 2 hours to block nonspecific binding of antibodies, followed by overnight incubation in primary antibodies diluted 1:1000 at 4°C. The membranes were then washed 3 times with TBST and incubated for 1 hour with second antibody diluted 1:5000 at room temperature. Bound antibodies were detected with ECL-enhanced chemiluminescence (Amersham Biosciences) according to the manufacturer's protocols. Hyperfilm-ECL exposed to membranes for 1 minute was developed in an X-ray film processor. Band intensity (area X density) was measured by densitometry (model GS-700, Imaging Densitometer; Bio-Rad). Background intensity was subtracted from all calculated areas and we used the ratio of control group as 1 as described previously[[1](#_ENREF_1)].

**Evaluation of cell viability**

C**ell viability was assayed by using** 3-(4,5-dimethyl-2-thiazolyl)-2,5-diphenyl-2-H- tetrazolium bromide (**MTT) as described previously[**[**2**](#_ENREF_2)**].** Cells were seeded into **96-well plate** at the density of 10000/ml and incubated for 24 hours. After treatment, 10 μl MTT (5 mg/ml) was added into cultured medium in each well **for 2-4 hours** until purple precipitate is visible. **After removal of culture medium,** 75 μl dimethyl sulfoxide was added to each well and leave the cells at room temperature in the dark for 2 hours. The absorbance at 570 nm was recorded.

**Cell migrations**

Scratch test was applied to detect the migration of cells[[3](#_ENREF_3),[4](#_ENREF_4)]. When the cell growth reached 80% fusion, cell digestion was inoculated into 24 well plates with six duplicated wells for each group. A scratch was made in the well bottom by using a sterile 10 ml spear in cultured cells. The picture of cell migration was taken at day 0 and day 4 after scratch. The migration rate was calculated by counting the distance of cell migrations.

***In vitro* tube formation assay**

The tube formation was performed as the method described previously[[5](#_ENREF_5)]. Cultured HUVEC were seeded on cell culture dishes coated with growth factor reduced Matrigel (BD Biosciences) and cultured in MCDB 133 medium containing 0.5% FCS. After 24 hours, the medium was removed and the cells were fixed with 4% paraformaldehyde. Photographs were taken through a microscope (Olympus, Tokyo, Japan). The capillary tube area was quantified per square micrometer using image analysis software (Image J Corporation).

**The *in vitro* model of oxygen glucose deprivation (OGD)**

OGD was carried out as described previously[[6](#_ENREF_6)]. Briefly, cells were placed in a 37°C anaerobic chamber with O_2_ tension at 1.5%. Cells were washed 3 times and incubated with glucose-free balanced salt solution containing 125 mM NaCl, 5 mM KCl, 1.2 mM NaH_2_PO_4_, 26 mM NaHCO_3_, 1.8 mM CaCl_2_, 0.9 mM MgCl_2_, and 10 mM HEPES that had been deoxygenated by 10 min of sparing with nitrogen. Control wells were washed and incubated with standard (non-deoxygenated) balanced salt solution containing 5 mM glucose. pH was maintained at 7.2-7.4 throughout

**eNOS activity assay**

eNOS activity was monitored by L-[^3^H]citrulline production from L-[^3^H]arginine as described previously[[7](#_ENREF_7)]. Briefly, protein samples were incubated in reaction buffer [1 mM L-arginine/100 mM NADPH/1 mM tetrahydrobiopterin/0.2 µCi of L-[^3^H]arginine (>66 Ci/mmol) per reaction] for 15 min at 37°C, separated by Dowex-50W ion-exchange chromatography in 20 mM HEPES (pH 5.5), 2 mM EDTA, and 2 mM EGTA, and the flow-through was used for liquid scintillation counting.

**Detection of NO**

NO production in culture cells was detected using the fluorescent probe DAF as described previously[[8](#_ENREF_8)]. In brief, before the end of treatment, 10 µM DAF was added to the medium and incubated for 30 min at 37°C, then washing with PBS. The DAF fluorescent intensity was recorded by fluorescent reader at the wave of excitation (485 nm) and emission (545 nm).

**RNA-immunoprecipitation (RIP) assay**

The Magna RIP kit was used for RNA IP assay as described previously[[9](#_ENREF_9)]. Briefly, whole-cell lysates were incubated at 4°C overnight with magnetic protein A/G beads pretreated with 5 μg IgG or Akt antibody. Beads were washed and incubated with proteinase K buffer, then RNA was isolated from immunoprecipitates, and cDNA was synthesized.

**Animals and experimental protocols**

Male C57B16 mice (8-12 weeks old, 25 ± 5 g) were purchased from Hua-Fu-Kang Animal Company (Beijing, China). All animals were housed in temperature-controlled cages with a 12-hour light-dark cycle. Mice received tail vein injection of adenovirus expressing control shRNA or lncRNA-ANRIL shRNA. For infection, virus was injected in 100 μl of PBS containing 7.6 X 10^7^ IFUs of loaded virus per. One week after virus infection, echocardiography was performed to assess heart function followed by MI surgery. At the 14^th^ postoperative day, echocardiography was performed again to assess heart functions. At the end of experiment, all mice were sacrificed under anesthesia by intraperitoneal injection of 0.8% pentobarbital sodium (60 mg/kg). This study was carried out in strict accordance with the recommendations in the Guide for the Care and Use of Laboratory Animals of the National Institutes of Health. The animal protocols were reviewed and approved by the Animal Care and Use Committees of Central South University.

**Myocardial infarction**

As described previously[[10](#_ENREF_10)], we followed these steps to establish MI model in mice. (1) Sterilize surgical instruments with a dry bead sterilizer (Germinator 500). (2) All mice (aged 8-12 weeks) were anesthetized with 2-3% isoflurane inhalation in an inducing chamber. (3) Once anesthetized, the mouse is removed from the inducing chamber to the surgical board, immobilized with tape, and continuously anesthetized with 2% isoflurane via coaxial breathing apparatus but not ventilated. (4) Remove the fur with a standard depilatory (e.g., Nair) and clean the skin with water and then betadine and alcohol pads. In order to perform this procedure more efficiently, the step of fur-removing could be done earlier. (5) Two small incisions (0.5 cm long) are made on the left and right chest skin with the scissors to expose the 3rd intercostal space. (6) Echocardiography is performed using a VEVO 2100 imaging system (Visual Sonics Inc., Toronto, Canada) with a 30 MHz phased array transducer and a frame rate of 235/s. The echocardiography probe (MS-400) is placed perpendicular to the sagittal plane of the chest within the 3rd intercostal space, imaging the left ventricle (LV) short axis. (7) A small straight needle (0.2 mm in diameter) was inserted at the costal angle of the superior margin of the 3^rd^ rib in the left chest. Under the guide of ultrasound, the heart is punctured in the inferior of left anterior descending coronary artery (LADCA) by a 8-0 silk suture attached to the needle. The needle is coming out of skin from the right chest. (8) Then, the needle is inserted back from the right to of the left. When the needle passes through the heart, it goes through above LADCA under ultrasound and came out the skin from the same site in the left chest. (9) Once a loose knot is made, the needle is inserted back from the left to the right in the chest. The LADCA is now located inside of the knot. (10) Ligation of LADCA by pulling the two ends of the suture carefully. The ischemia was confirmed by the elevation of ST segment recorded by the echocardiography imaging system during the surgery. The knot is readily visible under ultrasound. (11) The mouse is then allowed to breathe room air and monitored on a heating blanket during the recovery period, which is generally complete within 3-5 min. (12) The sham group undergoes the same surgical procedures except that the LADCA is not occluded. (13) One dose of buprenorphine (0.1 mg/kg) is administered subcutaneously (s.c.) immediately after the incision is closed.

**Echocardiography**

Echocardiography was performed as described previously[[11](#_ENREF_11)]. Ultrasonography with standard parasternal and apical views was performed in mice in the left lateral recumbent position. We obtained high-quality 2-D images including apical (4-chamber, 2-chamber and long-axis) and short-axis views (mitral annulus, papillary muscle and apex) with use of a 2.0-4.0 MHz transducer at a frame rate of 60 to 100 frames/sec and 3 consecutive cardiac cycles during breath hold. Images were digitized in cine-loop format and stored.

**Capillary density**

Histological analysis was assessed in perfusion/fixed hearts collected from mice at 14 days after surgery as described previously[[12](#_ENREF_12)]. The right atrium was then cut and the myocardial vasculature was perfused, followed by 10 min perfusion with 10% formalin. The hearts were harvested and fixed in 4% formalin for 24 hours. The formalin-fixed tissues were embedded in paraffin wax and cut into 5 μm sections. For the measurement of capillary density (counts/mm^2^), we performed immunohistochemical analysis of CD31. Transverse sections of the short axis of the left ventricle per sample were used in this analysis. Five fields on the slide were randomly chosen for counting the stained capillaries in the border zone between infarcted area and non-infarcted area at 400X magnification. All stained capillaries were counted, and the density was expressed as number per scope as described previously[[13](#_ENREF_13)].

**Statistical analysis**

All quantitative results are expressed as mean ± SEM. The normal distribution of data was tested by the Kolmogorov-Smirnov test before statistical comparisons, and the normality/equal variance was tested to determine whether ANOVA was appropriate. Multiple comparisons were analyzed with a one-way ANOVA followed by Tukey *post-hoc* tests or Bonferroni *post-hoc* analyses. Comparisons between two groups were analyzed by unpaired Student's *t* tes. Statistical analyses were conducted using GraphPad Prism 6.0 or IBM SPSS statistics 20.0. A two-sided *P*-value < 0.05 was considered significant.

**References**

1. **Wang S, Xu J, Song P, Viollet B, Zou MH.** In vivo activation of AMP-activated protein kinase attenuates diabetes-enhanced degradation of GTP cyclohydrolase I. *Diabetes*. 2009; 58: 1893-901.

2. **Zhao H, Zhang T, Xia C, Shi L, Wang S, Zheng X, Hu T, Zhang B.** Berberine ameliorates cartilage degeneration in interleukin-1beta-stimulated rat chondrocytes and in a rat model of osteoarthritis via Akt signalling. *Journal of cellular and molecular medicine*. 2014; 18: 283-92.

3. **Baggott RR, Alfranca A, Lopez-Maderuelo D, Mohamed TM, Escolano A, Oller J, Ornes BC, Kurusamy S, Rowther FB, Brown JE, Oceandy D, Cartwright EJ, Wang W, Gomez-del Arco P, Martinez-Martinez S, Neyses L, Redondo JM, Armesilla AL.** Plasma membrane calcium ATPase isoform 4 inhibits vascular endothelial growth factor-mediated angiogenesis through interaction with calcineurin. *Arterioscler Thromb Vasc Biol*. 2014; 34: 2310-20.

4. **Miyake H, Maeda K, Asai N, Shibata R, Ichimiya H, Isotani-Sakakibara M, Yamamura Y, Kato K, Enomoto A, Takahashi M, Murohara T.** The actin-binding protein Girdin and its Akt-mediated phosphorylation regulate neointima formation after vascular injury. *Circ Res*. 2011; 108: 1170-9.

5. **Nakamura M, Mie M, Mihara H, Nakamura M, Kobatake E.** Construction of multi-functional extracellular matrix proteins that promote tube formation of endothelial cells. *Biomaterials*. 2008; 29: 2977-86.

6. **Liu C, Liang B, Wang Q, Wu J, Zou MH.** Activation of AMP-activated protein kinase alpha1 alleviates endothelial cell apoptosis by increasing the expression of anti-apoptotic proteins Bcl-2 and survivin. *J Biol Chem*. 2010; 285: 15346-55.

7. **Davis BJ, Xie Z, Viollet B, Zou MH.** Activation of the AMP-activated kinase by antidiabetes drug metformin stimulates nitric oxide synthesis in vivo by promoting the association of heat shock protein 90 and endothelial nitric oxide synthase. *Diabetes*. 2006; 55: 496-505.

8. **Thomas S, Kotamraju S, Zielonka J, Harder DR, Kalyanaraman B.** Hydrogen peroxide induces nitric oxide and proteosome activity in endothelial cells: a bell-shaped signaling response. *Free Radic Biol Med*. 2007; 42: 1049-61.

9. **Bierhoff H.** Analysis of lncRNA-Protein Interactions by RNA-Protein Pull-Down Assays and RNA Immunoprecipitation (RIP). *Methods Mol Biol*. 2018; 1686: 241-50.

10. **Sun Q, Wang KK, Pan M, Zhou JP, Qiu XT, Wang ZY, Yang Z, Chen Y, Shen H, Gu QL, Fang LH, Zhang GG, Bai YP.** A minimally invasive approach to induce myocardial infarction in mice without thoracotomy. *Journal of cellular and molecular medicine*. 2018; 22: 5208-19.

11. **Yang J, Liu X, Jiang G, Chen Y, Zhang Y, Zhang M.** Two-dimensional strain technique to detect the function of coronary collateral circulation. *Coron Artery Dis*. 2012; 23: 188-94.

12. **Li CC, Qiu XT, Sun Q, Zhou JP, Yang HJ, Wu WZ, He LF, Tang CE, Zhang GG, Bai YP.** Endogenous reduction of miR-185 accelerates cardiac function recovery in mice following myocardial infarction via targeting of cathepsin K. *Journal of cellular and molecular medicine*. 2019; 23: 1164-73.

13. **Grundmann S, Hans FP, Kinniry S, Heinke J, Helbing T, Bluhm F, Sluijter JP, Hoefer I, Pasterkamp G, Bode C, Moser M.** MicroRNA-100 regulates neovascularization by suppression of mammalian target of rapamycin in endothelial and vascular smooth muscle cells. *Circulation*. 2011; 123: 999-1009.

**
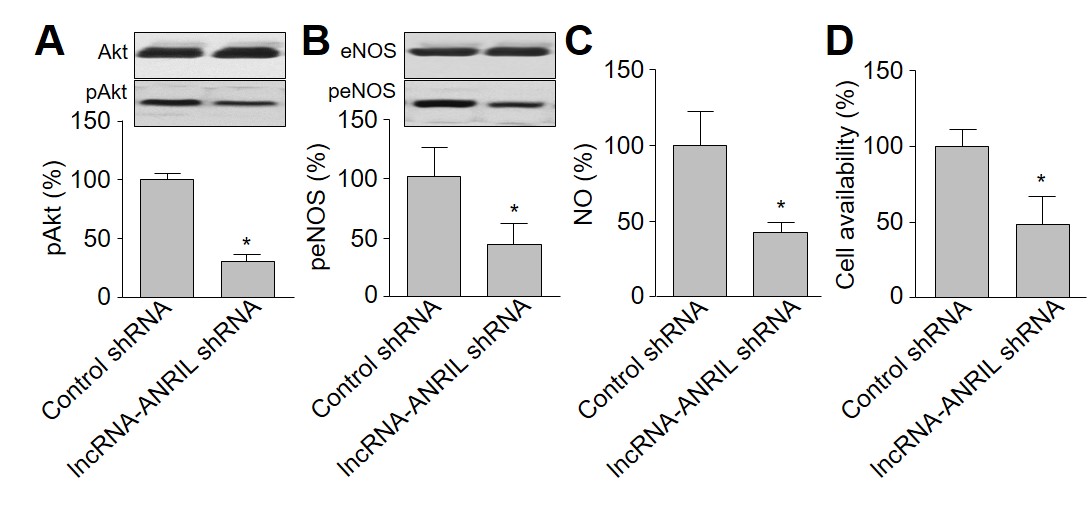
 Figure S1. Gene knockdown of lncRNA-ANRIL reduces the phosphorylated levels of Akt and eNOS proteins, decreases NO productions, and impaired cell availability in HUVECs.** Cultured HUVECs were infected with adenovirus expressing lncRNA-ANRIL shRNA or control shRNA for 48 hours. (**A** and **B**) Total cell lysates were subjected to perform western blot to meassure the phosphorylated levels of Akt in **A** and eNOS in **B**. (**C**) Intracellular NO productions were determined by DAF fluorescence. (**D**) Cell viability was meassured by MTT assay. N is 5 in each group. **P*<0.05 vs control shRNA.

**
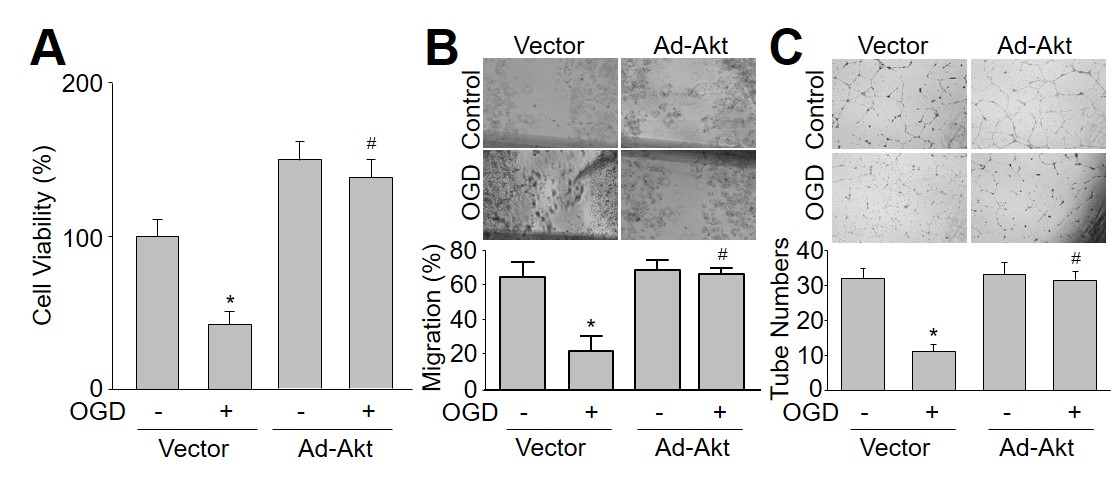
Figure S2. Overexpression of Akt rescues OGD-induced impairments of cell migration and tubulogenesis in HUVECs**. Cultured HUVECs were infected with adenovirus expressing Akt cDNA for 48 hours and then treated with OGD for 6 hours. (**A**) Cell viability was meassured by using MTT assay. (**B**) Cell migration was determined by scratch test. Migration rate was calculated in the 3^rd^ day after scratch. (**C**) Tubulogenesis of HUVECs was determined by tube formation test. N is 5 in each group. ^*^*P*<0.05 vs vector alone. ^#^*P*<0.05 vs vector plus OGD.

**
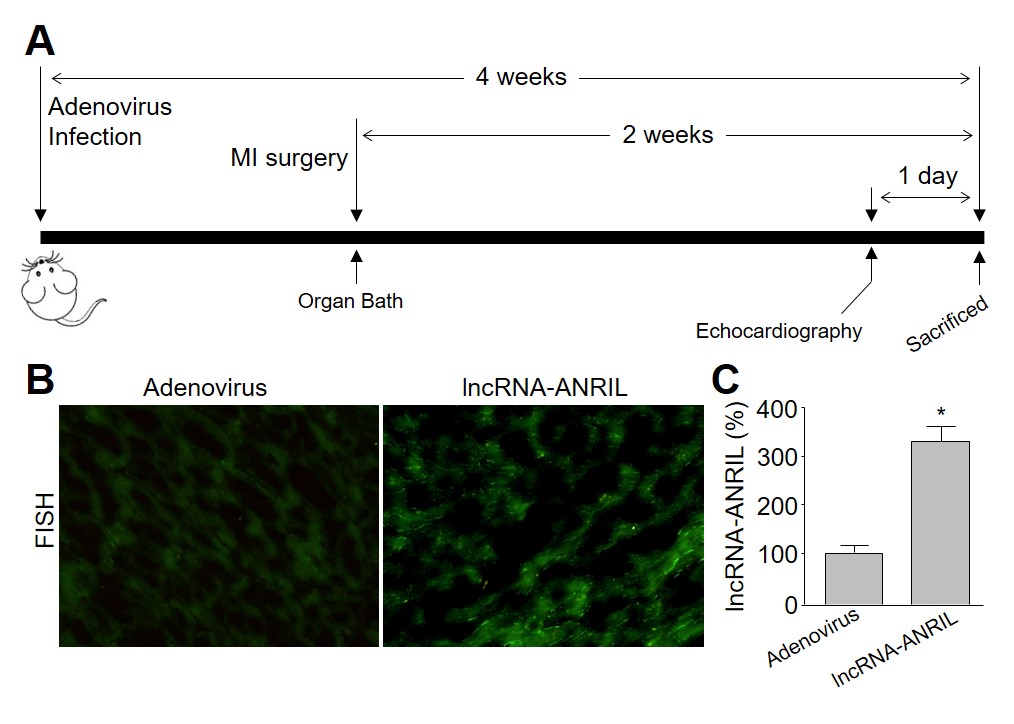
 Figure S3. Adenovirus-mediated knockdown of lncRNA-ANRIL promotes angiogenesis in postischemic myocardium in mice with MI.** (**A**) The protocol and experimental designs were described in Supppl. Method and illustrated. (**B** and **C**) The efficiency of adenovirus-mediated *in vivo* overexpression of lncRNA-ANRIL was determined by FISH in **B** and real-time PCR in **C**. N is 10 in each group. ^*^*P*<0.05 vs adenovirus.

**
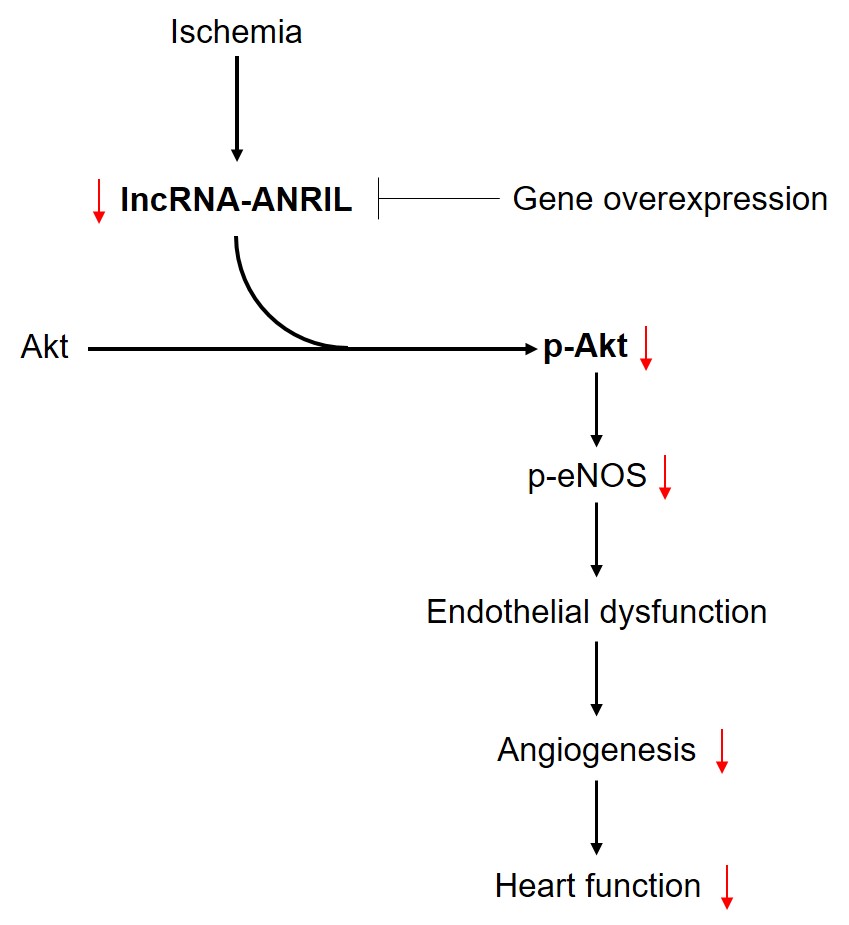
**

**Figure S4. Proposed mechanism by which lncRNA-ANRIL improves cardiac functions**. Ischemia downregulates lncRNA-ANRIL gene expression, leading to the decreased affinity of lncRNA-ANRIL to Akt protein. As a result, Akt phosphorylation is decreased and the Akt/eNOS signaling is dysfunctional. In this way, overexpression of lncRNA-ANRIL normalizes Akt/eNOS pathway to promote angiogenesis and to improve heart functions after MI.
